# Recent Advances in Antimicrobial Peptide Hydrogels

**DOI:** 10.3390/ijms24087563

**Published:** 2023-04-20

**Authors:** Aryanna Copling, Maxwell Akantibila, Raaha Kumaresan, Gilbert Fleischer, Dennise Cortes, Rahul S. Tripathi, Valerie J. Carabetta, Sebastián L. Vega

**Affiliations:** 1 Department of Molecular & Cellular Biosciences, Rowan University, Glassboro, NJ 08028, USA; coplin22@students.rowan.edu; 2 Department of Biomedical Sciences, Cooper Medical School of Rowan University, Camden, NJ 08103, USA; akanti18@students.rowan.edu (M.A.); fleischeg0@rowan.edu (G.F.); cortesd@rowan.edu (D.C.); tripat45@rowan.edu (R.S.T.); 3 Department of Biomedical Engineering, Rowan University, Glassboro, NJ 08028, USA; kumare76@students.rowan.edu; 4 Department of Orthopedic Surgery, Cooper Medical School of Rowan University, Camden, NJ 08103, USA

**Keywords:** antibiotic resistance, biofilm, click chemistry, photopolymerization, AMP-releasing, self-assembling, hydrogel, antimicrobial peptide

## Abstract

Advances in the number and type of available biomaterials have improved medical devices such as catheters, stents, pacemakers, prosthetic joints, and orthopedic devices. The introduction of a foreign material into the body comes with a risk of microbial colonization and subsequent infection. Infections of surgically implanted devices often lead to device failure, which leads to increased patient morbidity and mortality. The overuse and improper use of antimicrobials has led to an alarming rise and spread of drug-resistant infections. To overcome the problem of drug-resistant infections, novel antimicrobial biomaterials are increasingly being researched and developed. Hydrogels are a class of 3D biomaterials consisting of a hydrated polymer network with tunable functionality. As hydrogels are customizable, many different antimicrobial agents, such as inorganic molecules, metals, and antibiotics have been incorporated or tethered to them. Due to the increased prevalence of antibiotic resistance, antimicrobial peptides (AMPs) are being increasingly explored as alternative agents. AMP-tethered hydrogels are being increasingly examined for antimicrobial properties and practical applications, such as wound-healing. Here, we provide a recent update, from the last 5 years of innovations and discoveries made in the development of photopolymerizable, self-assembling, and AMP-releasing hydrogels.

## 1. Introduction

### 1.1. Medical Device Infections Are a Global Concern

Surgically implanted medical device use has greatly increased over the past 50 years, improving the quality of life of millions of people worldwide. Advancements in the types of biomaterials available has expanded and improved medical devices, such as catheters, stents, pacemakers, prosthetic joints, and orthopedic devices (reviewed in [1]). The introduction of a foreign material into the body comes with an inherent risk of microbial colonization and subsequent infection [2,3]. The infection can occur during the actual surgical procedure or post-operatively, with the source of infection frequently being the improper sanitization of surgical equipment, the hands of healthcare workers, or the colonization of the patient [4]. Infections of implanted medical devices are especially challenging to treat, due the presence of bacterial biofilms [5]. Bacteria can exist in a free-living, planktonic state or as part of a multicellular biofilm community. When a free-living bacterium attaches to a surface, it switches to a sessile lifestyle and there is a drastic change in gene expression. In particular, the genes required to produce extracellular polymeric substance (EPS) are induced. The biofilm matrix is composed of mostly polysaccharides, proteins, and extracellular DNA, which encases the growing colony in a slime layer that protects the bacteria against environmental insults [6,7,8].

In the natural environment, nearly every bacterial species can assemble into biofilms [9]. From a clinical standpoint, biofilms protect bacteria from host immune defenses and antibiotics, making them difficult to eradicate [10,11,12]. Clinically, biofilm-based infections of surgically implanted devices often lead to device failure, which then requires treatment with high doses of antibiotics, followed by the removal and replacement of the infected device. This puts the patient in need of additional, potentially risky surgeries and increases the risk of mortality [13]. Current interventions to address device-associated infections are also costly and often ineffective due to the prevalence of antibiotic-resistant strains and the increased risk of the reinfection of the new implant [8,10]. As the biofilm matures, essential nutrients become limited and toxic byproducts accumulate, causing the surface layer of the bacteria to revert to a planktonic lifestyle and travel to other body sites to begin the process again. Thus, bacterial biofilms are also a reservoir for chronic infections [6,8]. Biofilms are associated with up to 80% of chronic bacterial infections [14].

### 1.2. Hydrogels as Promising Additions to Our Antimicrobial Arsenal

To overcome the problem of chronic infections, antimicrobial materials are increasingly being researched and developed. These materials are typically polymers, ceramics, metals, or composites, which have microbicidal activity against bacteria, fungi, viruses, or a combination of the three. One such material being extensively studied is hydrogel (reviewed in [15]). Hydrogels are soft biomaterials consisting of highly hydrated polymeric networks that have been tailored with various moieties for applications as antibacterial, tissue-regenerating, and drug-releasing materials [16,17]. As hydrogels are customizable, many different antimicrobial agents have been incorporated or tethered to them. For example, inorganic molecules, such as metal ions and metallic oxides, have been explored. Common metal ions include silver, gold, and copper and the most common metal oxides are zinc, titanium, and nickel [18]. It is generally believed that metal-loaded hydrogels kill bacteria by disrupting and destroying the bacterial cell wall. Silver-loaded hydrogels have broad-spectrum activity, as they are efficient at killing both Gram-negative and Gram-positive bacteria [19,20,21]. While silver ions are promising antimicrobial agents, their cytotoxicity and interactions with serum proteins, such as albumin, must be considered [22]. While other metals, such as cobalt, copper, and gold, have been studied as alternative antimicrobial agents, these metals have not been sufficiently explored [15]. As with silver ions, the toxicity and clearance of these metals is a significant concern. Metal oxide-loaded hydrogels kill bacteria through the generation of large amounts of reactive oxygen species following exposure to light [23]. Metal oxide-loaded hydrogels, the most widely used being zinc oxide (ZnO), are highly effective and non-toxic to human cells at low concentrations [24,25,26]. While inorganic agent-loaded hydrogels have many promising, desirable properties, such as broad-spectrum activity and temperature stability, they have low biocompatibility, making their use in practice a challenge.

As an alternative to metal oxide-laden hydrogels, antibiotic-tethered hydrogels have also been studied. Tethering antibiotics allows for a lower dosage to be used, which in theory should lessen the risk of antibiotic resistance [27]. The three most studied antibiotics used for this application are ciprofloxacin, gentamicin, and vancomycin. The fluoroquinolone ciprofloxacin has a broad spectrum of activity. Ciprofloxacin-loaded hydrogels have excellent antimicrobial properties, with low cytotoxicity and no hemolytic activity [28,29,30]. Gentamicin is an aminoglycoside antibiotic with broad spectrum activity but carries a risk of nephrotoxicity. Gentamicin-tethered hydrogels allow for the local administration of the antibiotic, which overcomes the other issue with this drug, namely achieving an acceptably high serum concentration [31,32,33]. Vancomycin is widely used in clinical practice and often considered a last-line drug for many Gram-positive infections, including *Enterococcus* and *Staphylococcus* species. Vancomycin-loaded hydrogels have shown promise in protecting against surgical site infections [34]. However, the spread and emergence of drug-resistant infections has dampened the enthusiasm for antibiotic-tethered hydrogels.

### 1.3. Antimicrobial Peptides as a Potential Solution to the Antibiotic Resistance Problem

The overuse and misuse of antibiotics has led to the rise and spread of drug-resistant infections. In 2019, 1.27 million deaths were attributed to drug-resistant bacteria worldwide [35]. According to a comprehensive antimicrobial resistance report, it was estimated that this number would increase to 10 million deaths per year worldwide by 2050 if new solutions to combat bacterial drug resistance are not identified and implemented [36]. As antibiotic use has increased steadily in the past 50 years, we are now seeing the emergence of multidrug-resistant strains which are non-susceptible to three or more standard-of-care antibiotics. Recently, the emergence of pan-drug resistant strains, which are resistant to all standard-of-care antibiotics, have been reported [37,38,39]. Due to the increased prevalence of antibiotic resistance, antimicrobial peptides (AMPs) are being increasingly explored as alternative antimicrobial agents. AMPs are host defense peptides and are found in all domains of life, but the majority studied are eukaryotic in origin [40]. They are a diverse class of short amino acid sequences that are effective as broad-spectrum antibacterial agents and in mitigating bacterial biofilms [41,42]. In general, AMPs are cationic peptides and contain both hydrophobic and hydrophilic components. Most of the identified AMPs assemble into α-helical structures and disrupt the bacterial membrane by pore formation, leading to cell death [40,43]. However, other structures, such as β-sheets, are commonly observed as well [44]. While membrane disruption is a common mechanism of action, AMPs may also target nucleic acid synthesis, enzymatic activities, and cell wall biogenesis [45]. Resistance to AMPs is thought to arise and spread more slowly, which makes them attractive as novel therapeutics [46,47]. AMP-tethered hydrogels are being increasingly examined for antimicrobial properties and practical applications. Here, we provide a review of the last 5 years of innovations and discoveries made in the development of AMP-loaded hydrogels. We begin with a discussion of new developments with photopolymerizing hydrogels. Next, we discuss advancements made with self-assembling and AMP-releasing hydrogels. Finally, we end with a brief discussion of remaining challenges and outlook for AMP-loaded hydrogel technology towards clinical practice.

## 2. Photopolymerizing AMP Hydrogels

### 2.1. Recent Updates Using Chitosan and Polyethylene Glycol Backbones

**Figure 1:**
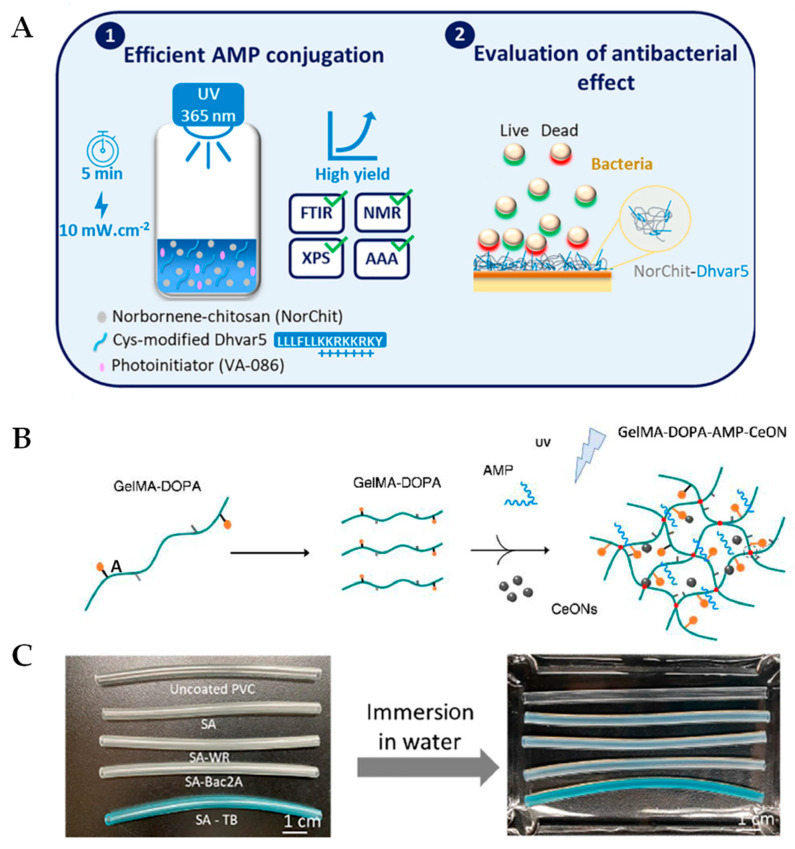
New photopolymerizable, antimicrobial hydrogel formulations created using UV light. (**A**) Norbornene-chitosan (NorChit) was exposed to the photoinitiator VA-086 and UV light (365 nm) at 10 mW/cm^2^ for 5 min to initiate the conjugation of cysteine-modified Dhvar5 (NorChit-Dhvar5). The polymers were confirmed and analyzed by Fourier transform infrared spectroscopy (FTIR), proton nuclear magnetic resonance (NMR), X-ray photoelectron spectroscopy (XPS), and amino acid analysis (AAA). Next, the antimicrobial properties of the NorChit-Dhvar5 films were analyzed and showed a reduction of total adhered and enhanced killing of *S. epidermidis* and *P. aeruginosa*. Reprinted with permission from Ref. [51]. 2022, American Chemical Society. (**B**) GelMA-DOPA polymers were mixed with antimicrobial peptide (AMP) and cerium oxide nanoparticles (CeONs) and exposed to UV light to initiate hydrogel formation. A sprayable antimicrobial and reactive oxygen species (ROS)-scavenging hydrogel was formed. Reprinted with permission from Ref. [56]. 2021, Elsevier. (**C**) Images of various PVC tubes, from top to bottom: uncoated tube, hydrogel (SA) coating, SA-WR, SA-Bac2A, and SA-TB (SA hydrogel coating stained with toluidine blue to demonstrate the uniform and complete coating of the surface). Reprinted with permission from Ref. [65]. 2021, American Chemical Society.

The synthesis of hydrogels is typically performed through photopolymerization using visible or ultraviolet (UV) light in the presence of a photoinitiator [48]. Photopolymerization reactions are rapid and step-growth reactions between thiols and vinyl (ene) groups yield highly specific click reactions that can be used to form hydrogels functionalized with thiolated molecules, such as peptides [49,50]. For instance, Alves and coworkers developed AMP-laden chitosan hydrogels by first conjugating norbornene groups into chitosan (NorChit), followed by clicking NorChit with thiolated Dhvar5 AMPs using a light-mediated step-growth reaction (NorChit-Dhvar5) [51]. The Dhvar5 (LLLFLLKKRKKRKY) AMP was chosen due to previously established antimicrobial properties [52,53]. Dhvar5-NorChit thin films were then prepared by spin coating NorChit and NorChit-Dhvar5 solutions onto gold (Au) substrates, as had been carried out previously [53]. These films demonstrated up to a 35% reduction in *Staphylococcus epidermidis* adhesion and increased the killing of *Pseudomonas aeruginosa* in comparison to unmodified chitosan films (Figure 1A). These films are also biocompatible, as demonstrated by toxicity assays performed on human neonatal dermal fibroblasts. This coating strategy can be used to create antibacterial biomaterials, and excitingly, there were remaining pendant norbornene groups, which could be used to add more Dhvar5 or any thiolated AMP to further increase antimicrobial properties.

In a recent study by De Zoysa et al., battacin lipopeptide hydrogels were synthesized by covalently linking N-terminal cysteine containing lipopeptides onto polyethylene glycol (PEG) macromers using a thiol-ene click chemistry reaction [54]. Battacin was chosen due to its potent in vitro and in vivo antibacterial activity against *P. aeruginosa* and *Staphylococcus aureus* [55]. The battacin-loaded hydrogels (0–10 wt%) were formed by mixing a tetra-branched thiol crosslinker, a diacrylate spacer, PEG, and a photoinitiator. Gelation was initiated by photopolymerization under UV irradiation. The hydrogels were prepared in either methanol or water, and the ones prepared in methanol had better antibacterial and antibiofilm activity. A minimum peptide content of 0.5 wt% with respect to polymer content was required to successfully inhibit planktonic bacterial growth and disperse the mature biofilms of *P. aeruginosa* and *S. aureus*. The hydrogels displayed excellent cytocompatibility and were not hemolytic. Together, these properties make these hydrogels good candidates for clinical applications, such as wound dressings to combat skin infections.

### 2.2. Development of Photopolymerizable Hydrogels for Clinical Applications

Towards developing hydrogels for wound dressing applications, Cheng and colleagues created a sprayable hydrogel to alleviate the impact of wound infection by using cerium oxide nanoparticles (CeONs), AMPs, and gelatin methacrylate (GelMA) as the photopolymerizable hydrogel matrix [56]. The hydrogel wound dressing was made from GelMA conjugated with dopamine (GelMA-DOPA) by (N-(3-(dimethylamino)-propyl)-N′-ethylcarbodiimide hydrochloride (EDC) and N-hydroxysuccinimide (NHS) coupling. The GelMA-DOPA hydrogels were formed by addition of the photoinitiator Irgacure 2959, CeONs, and the AMP HHC-36 (KRWWKWWRR) as well as exposure to UV light for 30 s to produce AMP-CeONs, which were loaded to GelMA-DOPA hydrogels (Figure 1B). HHC-36 exhibited nearly complete bactericidal activity against both Gram-negative and Gram-positive pathogenic bacteria [57]. The AMP-loaded GelMA-DOPA hydrogels displayed the complete bacterial killing of *S. aureus*, *S. epidermidis*, *P. aeruginosa*, and *Escherichia coli* over the course of 24 h. In addition, the GelMA-AMP-CeONs combination showed the most promising outcomes of enhancing healing speed and the promotion of newly formed skin in mouse wound models over 14 days, partly by the reactive oxygen species (ROS) scavenging property of the CeONs. Hydrogel-treated wounds also resulted in significant native type I and type III collagen deposition, which plays a critical role in wound-healing [58]. The notable advantages of using the AMP-CeONs-loaded GelMA-DOPA hydrogels as a sprayable wound dressing are the pliability, adhesiveness, antimicrobial activity, and skin-remodeling abilities of these novel materials.

Hydrogels have high potential for use as wound dressings, due to their ability to be tailored to mimic the composition and physiochemical properties of the human extracellular matrix. They tend to be highly biocompatible and biodegradable in vivo [59]. However, hydrogels alone tend to be mechanically weak, exhibit poor adhesion to wet tissues, and have limited antimicrobial activity [60,61]. Annabi et al. developed novel hydrogels for sutureless wound closures that address these shortcomings [62]. The backbone of the hydrogel was a combination of GelMA and methacrylol-substituted recombinant human tropoelastin (MeTro). The AMP Tet 213 (KRWWKWWRRC) was tethered to MeTro/GelMA composite hydrogels using visible light-induced crosslinking. Tet 213 has broad spectrum activity and was previously tethered to titanium coatings to inhibit biofilm formation of *S. aureus* in vitro [63]. Increasing the hydrogel polymer concentration resulted in increased adhesive strength, burst pressure, and wound-sealing capabilities compared to commercially available tissue adhesives. The Tet 213-loaded MeTro/GelMA hydrogels inhibited the bacterial colonization of methicillin-resistant *S. aureus* (MRSA) and *E. coli* to a similar extent as ZnO nanoparticles [64]. The Tet 213-loaded MeTro/GelMA hydrogels also supported the growth, spread, and proliferation of 2D surface-seeded and 3D encapsulated fibroblasts in vitro. The hydrogels induced minimal inflammatory responses and were shown to efficiently biodegrade when implanted subcutaneously in a murine animal model. Together, these properties make MeTro/GelMA-AMP hydrogels excellent alternatives to current sutureless wound closure approaches.

Towards developing novel antimicrobial coatings, Liu and coworkers designed an AMP-loaded hydrogel coating, with antibacterial, antithrombotic, and antifouling properties [65]. The hydrogel was composed of sulfobetaine methacrylate (SBMA) and acrylic acid (AAc), which have good hemocompatibility. The hydrogel (SA) was grafted on the inside surface of a polyvinyl chloride (PVC) tube following UV irradiation for one hour. The AMPs WR (WRWRWR-NH_2_) or Bac2A (RLARIVVIRVAR-NH_2_) were embedded using EDC/NHS chemistry to form SA-WR and SA-Bac2A hydrogels (Figure 1C). The cationic AMP bactenecin is one of the smallest AMPs and its derivative Bac2A has similar Gram-negative coverage but improved Gram-positive activity [66]. WR is an ultrashort AMP, which, when loaded into nanoparticles, displays excellent antimicrobial properties against *S. aureus* [67]. Few platelets aggregated on the surface and the hemolysis ratios of both hydrogels were less than 5%, revealing good hemocompatibility. The SA-Bac2 and SA-WR hydrogels completely prevented *E. coli* and *S. aureus* from growing on the hydrogel surface. After 4 weeks of storage at 37 °C, SA-Bac2 still had antimicrobial activity, but SA-WR lost some activity. This demonstrates the remarkable bioactive stability of the AMPs embedded in the SA-Bac2 hydrogels. Moreover, the embedded AMPs did not influence the antifouling or antithrombotic properties of the hydrogel coating. Excitingly, SA-Bac2 and SA-WR effectively killed *S. aureus* in a rat model with a catheter-induced infection. The SA-Bac2 hydrogel has the potential to be used in clinical practice as a coating to prevent bacterial infections and thrombosis formation for implanted medical devices that will contact blood.

Dental implants are highly prone to infection and Xie and coworkers developed a fine-tuned AMP adhesive system with improved mechanical properties and antimicrobial activity for application in the field of dentistry [68]. The AMP derivative GH12-M2 (GLLWHLLHHLLH_GSGGG_K) was conjugated to methacrylate (MA) monomers by stirring overnight at room temperature. The GH12-M2 peptide was selected for its activity against oral *Streptococci* [69,70]. The MA-AMP monomers were light-cured for 40 s using visible light. The MA-AMP conjugates had two different C-terminal spacers, GGG and SSSGGG. It was shown that the GGG spacer had superior antimicrobial activity against *Streptococcus mutans*, which may reflect conformational optimization for the improved activity of the AMP. These AMP-loaded hydrogels offer an attractive solution for dental composite restoration failure. Moreover, it was demonstrated that engineering and optimizing the spacer sequence provides the chance to fine-tune the activity of the peptide in the system. The optimization of the peptide linker sequence could be expanded to additional hydrogel applications, such as wound healing and infection prevention on medical devices.

## 3. Self-Assembling AMP Hydrogels

### 3.1. Thermosensitive Hydrogels

The main advantage of self-assembling hydrogels over photopolymerizable hydrogels is that they do not require light to form, thereby enabling their use for delivering therapeutics in a minimally invasive manner or in situations where light is not readily available. Hydrogel self-assembly can be triggered by ionic interactions, pH, or temperature [71]. Thermosensitive hydrogels can either transition from liquid to gel above or below a critical solution temperature [72]. For instance, gelatin is liquid at 37 °C and gels below 30 °C [73], whereas poly(N-isopropyl acrylamide) (PNIPAM) is liquid at room temperature and gels at physiologic temperatures [74]. With the aim of designing self-forming antimicrobial hydrogels that enhance wound-healing, Feng and coworkers reported the development of PNIPAM hydrogels containing a joint peptide consisting of an AMP RRWRVIVKW and RADA16 (Ac-RADARADARADARADA-NH_2_), termed RA-Amps [75]. The AMP used in this study was found to disrupt bacterial cell membranes and inhibit growth [76,77]. RADA-16 is an amphipathic peptide that self-assembles into a nanofibrous hydrogel [78] and RADA-16 was incorporated into their design as a barrier that prevents the overflow of fluid and cells into the wound site. To promote wound-healing, a mechano growth factor E peptide (MGF-E) was also included due to its well-established role in tissue repair [79]. The hydrogels were formed by dissolving hydrogel components separately, followed by mixing and heating solutions to 37 °C, resulting in rapid assembly (~23 s). The antibacterial properties of the hydrogels were confirmed by agar diffusion assays against *E. coli* and *S. aureus* strains treated with increasing concentrations of RA and RA-Amps. A concentration of 74 µM of RA or RA-Amps resulted in bactericidal activity with over 99% efficiency. PNIPAM/RA-Amps were also loaded with MGF-E peptides and using a murine wound model, the authors found increased the epithelialization, angiogenesis, and formation of fibrous collagen in comparison to commercial wound dressings. While the authors demonstrated the antimicrobial properties of RA-Amps in vitro, this was not confirmed in their in vivo wound model.

### 3.2. Peptide-Based Self-Assembling Hydrogels

**Figure 2:**
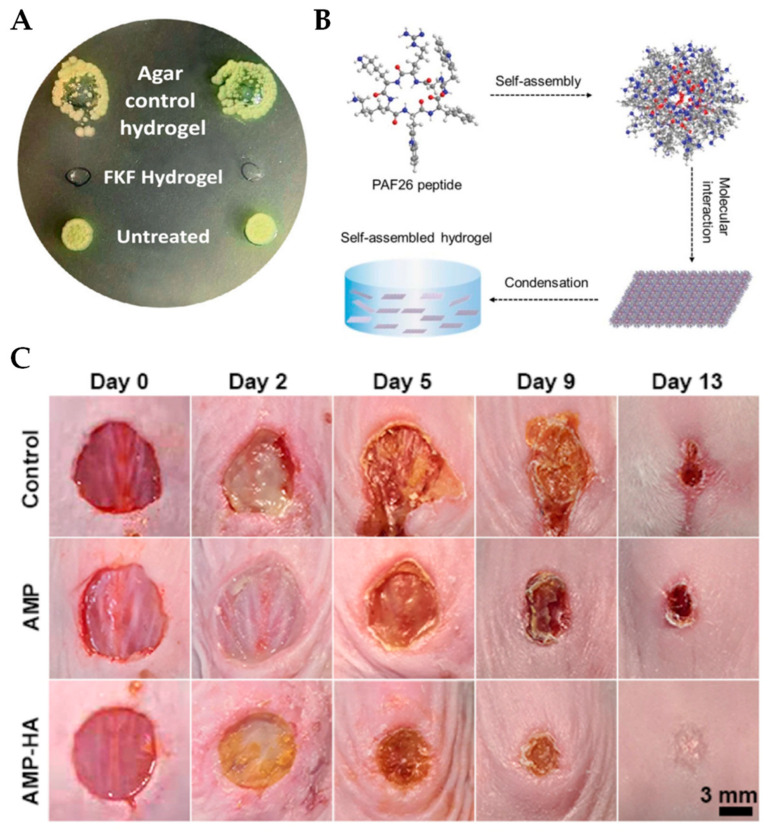
Self-polymerizing hydrogels are effective antimicrobial agents that can promote wound-healing. (**A**) *P. aeruginosa* spotted on an agar plate shows complete inhibition by the FKF hydrogel (center) compared to agar control hydrogels (top) and the untreated positive control (bottom). Reprinted with permission from Ref. [82]. Copyright 2021, Elsevier. (**B**) The proposed self-assembly mechanism of the PAF26 peptide. When the pH of the solution reached 7.5, the molecular stacking interactions lead to the formation of β-sheet structures that self-assemble into micelles. The micelles then are linked together into a chain-like structure, which, following condensation, leads to the formation of a hydrogel. Reprinted with permission from Ref. [89] Copyright 2019, The Royal Society of Chemistry. (**C**) In vivo wound-closure studies from *S. aureus*-infected mice wounds. Wounds were evaluated on days 0, 2, 5, 9, and 13 post-infection. The control group was untreated, while the AMP group was treated with soluble AMP, and the AMP-HA cohort was treated with AMP-loaded hydrogels. Reprinted with permission from Ref. [94]. Copyright 2021, American Chemical Society.

Self-assembling hydrogels can also be completely peptide-based, which form 3D fibrous networks via noncovalent crosslinking between peptides through ionic, hydrophobic, hydrogen, or π–π stacking interactions [80,81]. Certain amphipathic AMPs can form supramolecular hydrogels and self-assembly rates can be controlled by tuning the pH. For instance, Azoulay et al. designed self-forming AMP hydrogels containing the Phe–Lys–Phe (FKF) sequence [82]. FKF peptides are amphipathic and cationic, and these hydrogels were formed by dissolving FKF peptides in an acidic buffer. Self-assembly occurred within minutes and these hydrogels were found to have β-sheet structures, with H-bonding and π–π stacking, at a pH between 3.3 and 4.3. To evaluate their antimicrobial properties in vitro, an agar-based quantitative antibacterial efficacy assay was conducted, significantly reducing the presence of *E. coli*, *P. aeruginosa*, *Acinetobacter baumannii*, and *S. epidermidis* and demonstrating a broad spectrum of activity (Figure 2A). The antimicrobial properties were also evaluated in vivo by applying the hydrogel as a dressing to a wound inoculated with *P. aeruginosa* using a rat wound model. While this study reported a bacterial reduction of 50%, a delay in wound-closing was observed, which could be due to the acidic conditions required for the hydrogel to polymerize.

Amphipathic AMPs can also self-form within a physiological pH range, which can address biocompatibility concerns regarding AMP hydrogels that polymerize under acidic conditions. D-amino acids are more resistant to bacterial proteases [83] and some of these, including lysine, leucine, and valine, have antibacterial properties [84,85,86]. The KLVFFAK peptide (KK-11) contains a self-assembling motif [87], and Guo et al. synthesized this peptide using D-amino acids (KKd-11) to investigate its antimicrobial activity [88]. The KKd-11 hydrogels were formed with a peptide concentration of 10 mg/mL or higher within 10 min at room temperature. The peptide was first dissolved at a low pH and then the solution was made alkaline with NaOH resulting in hydrogel formation at a pH of 7 or higher. KKd-11 hydrogels prevented the biofilm formation of *E. coli* and *S. aureus* strains, and they killed the bacteria within the biofilms. Given their antimicrobial and inhibitory capabilities and their biocompatibility and higher resistance to proteolytic enzymes, the KKd-11 hydrogels have high potential to be used to prevent wound infections and prevent bacterial growth on medical devices.

In another study, Cao and coworkers synthesized a PAF26 AMP hydrogel that polymerizes at physiologic pH [89]. PAF26 is a hexapeptide with the sequence Ac-RKKWFW-NH_2_ that permeabilizes the cell wall, leading to eventual microbial killing [90]. PAF26 is amphipathic in nature and this peptide self assembles into a hydrogel triggered by pH. PAF26 was dissolved in water and the pH slowly increased until a pH of 7.5 was reached, which was determined to be the optimal pH for gelation. After holding at this pH for several minutes, a hydrogel was formed, and the resulting hydrogels were found to have β-sheet structures (Figure 2B). The PAF26-containing hydrogels were co-cultured with fungi or bacteria, and their antimicrobial activity was assessed by the determination of the zone of inhibition and by monitoring growth in the presence of the hydrogels. The PAF26-hydrogels inhibited the growth of *Candida albicans*, *S. aureus*, and *E. coli*. The hydrogels yielded a 100% killing efficiency against a variety of different pathogenic species. These PAF26 hydrogels have the potential for use in clinical practice; however, as the cytotoxicity was not determined, further optimizations may be required to minimize the concentration of PAF26 used that still contains excellent antimicrobial properties.

The research discussed thus far has relied on amphiphilic peptides that form β-sheet secondary structures and rupture cell walls [91]. Armed with this information, Adak et al. synthesized a novel antibacterial lipopeptide, containing palmitic acid-conjugated to a peptide with the sequence NAVSIQKKK (PA-NV) [92]. PA-NV was synthesized using solid-state peptide synthesis. The peptide hydrogel was prepared by dissolving 20 mg PA-NV in phosphate-buffered saline to yield a 2 wt% solution at pH 7.4. The solution was gently heated to dissolve the peptides, and after incubation for 30 min at room temperature, a homogenous PA-NV hydrogels self-assembled. Increasing the hydrogel concentration increased the number of three-dimensional cross-links in the nanofiber structure, which significantly facilitated gelation. The antibacterial activity, cytotoxicity, biocompatibility, and stability of the PA-NA hydrogels against enzymatic degradation were examined. PA-NV hydrogel surfaces were effective at preventing the growth of *E. coli* and *S. aureus* on inoculums of 10^5^–10^6^ cells/dm^2^. However, a larger inoculum of 2.0 × 10^8^ cells/dm^2^ or higher showed bacterial growth on the hydrogel surface. It was also found that PA-NV hydrogels are biocompatible, stable against enzymatic degradation by proteinase K, and non-cytotoxic against mammalian cells. This novel lipopeptide has both hydrophobic and hydrophilic domains. The cationic charge of PA-NV hydrogel makes it possible to interact with and disrupt the bacterial membrane, making this the most likely mode of action. The amphiphilic nature of the construct can form β-sheets, which aid in gelation, and produces a biocompatible soft material. These properties make the PA-NV hydrogels exciting and promising candidates for the development of such hydrogels for clinical applications. It will be interesting to see how these hydrogels perform in in vivo model systems.

### 3.3. AMP Self-Assembling Hydrogels with Multi-Functionality

Self-forming AMP hydrogels can also include additional networks to increase their functionality beyond antimicrobial activity. For example, hyaluronic acid (HA) is a component of the skin extracellular matrix (ECM) and has been shown to accelerate wound-healing [93]. In a study by Suo et al., the antimicrobial properties of the AMP KK(SLKL)_3_KK were studied with the inclusion of HA [94]. Hydrogels were formed by oxidizing HA, which was then dissolved and mixed with an AMP solution at a neutral pH. The antimicrobial properties were confirmed in vitro by co-incubating *S. aureus* and *E. coli* with soluble AMPs and the AMP-HA complexes at different concentrations. While a higher concentration of AMP-HA compared to AMP alone was required for complete killing, AMP-HA still had excellent antimicrobial activity. Further, the AMP-HA hydrogels greatly reduced healing times in a mouse wound model and promoted increased collagen deposition, wound epithelialization, and angiogenesis (Figure 2C). This hydrogel is promising for the possible development of an antimicrobial hydrogel to promote wound-healing in clinical practice.

Another example was reported by Yang and coworkers, who created a set of RADA16 hydrogels assembled with the AMP Tet 213 [95]. The RADA16-AMP hydrogels self-assembled with a 10 mg/mL RADA-AMP solution within 30 min at room temperature. The antimicrobial activity of RADA-AMP hydrogels compared to RADA-alone hydrogels against *S. aureus* was evaluated. It was found that the RADA-AMP hydrogels were able to continuously release AMPs over 28 days and had reduced *S. aureus* growth to a greater extent. After 28 days, AMPs remained in the hydrogel. In addition, the antimicrobial activity increased as the AMP concentration increased. An in vivo experiment was conducted using rabbits as a model system. A small quantity of bone marrow was extracted from the tibia and inoculated with 1.5 × 10^8^ bacteria/mL to establish an osteomyelitis model. After 4 weeks, RADA and RADA-AMP gels were injected into the same site and, 6 or 10 weeks later, were analyzed by micro-CT. The inflammation caused by the bacteria was limited to the injection site. Moreover, the combination of AMP with RADA16 resulted in new bone formation; the rabbits had more mature bone and greater bone regeneration compared to the controls and RADA-containing hydrogels. Given their antimicrobial efficacy, ability to promote osteogenesis, sustained release of AMPs, and biocompatibility, the RADA16-AMP hydrogels have a high potential to be used in patients to aid in the treatment of bone infections and the promotion of osteogenesis. Future work should concentrate on optimizing the concentration of Tet 213 for potency, the determination of the mechanism of action of RADA-AMP on microbial killing and osteogenesis, and possibly substituting other AMPs with superior antimicrobial or healing properties for other applications.

Zhou et al. studied the utility of the AMP, Jelleine-1 (J1), in combination with adenosine diphosphate (ADP). J1 is a natural, amphiphilic AMP that was isolated from the royal jelly of the honeybee, with a sequence of PFKLSLHL-NH_2_ [96]. ADP was found to be an important substance for platelet aggregation and coagulation, which was the rational for adding ADP to this hydrogel [97,98]. The J1-ADP hydrogel is formed by dissolving the J1 peptide into dimethyl sulfoxide to make a 100 mM stock solution [99]. An equal volume of ADP is added and stirred to allow for self-assembly into hydrogels. The antimicrobial, hemostatic, and anti-adhesion properties of this J1-ADP hydrogel were assessed. The J1-ADP hydrogel had good antimicrobial activity against some common bacterial and fungal pathogens, including *S. aureus*, *E. coli*, and *C. albicans*. It also had efficacy against MRSA strains. Postoperative peritoneal adhesion is a serious complication of abdominal surgery, which leads to severe abdominal pain, intestinal obstruction, organ disfunction, and additional complications [100,101,102]. The presence of the J1-ADP hydrogel in an in vivo mouse wound model demonstrated decreased intrabdominal adhesions and more efficient hemostasis, as determined by blood coagulation, platelet activation, and platelet adhesion assays. The injectable quality of these hydrogels makes them suitable for surgical contexts. Additionally, the anti-adhesive and hemostatic properties of this hydrogel also make it useful for application in large areas that are prone to adhesions. However, this study did not include an analysis of microbial burden with and without the J1-ADP hydrogel in vivo, which could demonstrate an additional infection prevention benefit. Nonetheless, self-forming hydrogels are promising materials for use in a wide variety of clinical applications.

## 4. AMP-Releasing Hydrogels

With the extended use of nanomaterials to deliver therapeutic agents to the target site, additional control over the release of the payload becomes possible. Any spatiotemporal control of drug release could improve efficacy, limit off-target effects, and minimize cytotoxicity. This technology has been applied in drug delivery systems for specific types of cancer cells [103,104]. Now, this same controlled-release technology is being developed for use with AMP delivery to sites of infection. There are three mechanisms of release that are being currently explored: temperature, pH, and enzymatic [105].

### 4.1. Temperature Controlled Release of AMPs

Rezaei and coworkers selected chitosan, which is a natural chitin-derived polysaccharide, for study because this material has many desirable properties, such as appropriate biodegradability, low toxicity, and antibacterial activity [106,107]. Thermo-responsive chitosan (TCTS) hydrogels were loaded with varying concentrations, from 0 to 16 µg/mL of the AMP piscidin-1 [108]. Piscidin-1 is a 22-residue, cationic peptide that was originally identified in the aquaculture striped bass. It has demonstrated potent antimicrobial activity against different bacterial and fungal species but is cytotoxic towards mammalian cells [109,110]. Due to the associated cytotoxicity, loading them into a hydrogel with controlled release is highly beneficial. The TCTS hydrogels were formed by dissolving 2% chitosan (*w*/*v*) in acetic acid and stirring for 24 h. To optimize the gelation time, the TCTS hydrogel was cross-linked at 4 °C with various concentrations of β-glycerophosphate disodium salt pentahydrate. It was found that the TCTS-40% β-glycerophosphate hydrogels had the shortest gelation time of 15 min at 37 °C. The formed TCTS hydrogels were loaded with 4, 8, and 16 μg/mL of piscidin-1. The antibacterial activity against susceptible and drug-resistant *A. baumannii* and the biocompatibility and release behavior of the AMP-TCTS hydrogels were determined. At the highest concentration of AMP tested (16 µg/mL), the hydrogels showed excellent bactericidal activity against the drug-resistant *A. baumannii* strains, while all concentrations were effective against the standard strain. On day 1, 40% of the piscidin-1 was released from the TCTS hydrogels and a controlled release of up to 7 days was observed. At day 7, 90% of the AMP had been released. Importantly, the AMP-TCTS hydrogels were not cytotoxic against human fibroblasts. The AMP-TCTS hydrogel represents a potential new, controlled delivery system of AMPs to the site of wound infections for the treatment of infections with drug-resistant pathogens. As piscidin-1 was previously reported to have hemolytic activity [111], it is important to determine this property of the AMP-TCTS hydrogels. In addition, in vivo animal wound models should also be analyzed to determine the antimicrobial and wound-healing properties of this system.

An extension of the temperature-controlled release of antimicrobial cargo is photothermal release. Moorcroft et al. developed a novel, nanoparticle-loaded hydrogel for the light-activated release and photothermal enhancement of AMPs [112]. To assemble the hydrogels, liposomes containing the AMP IK8 (IRIKIRIKCO NH_2_) were first prepared. IK8 is a β-sheet forming AMP with a potent broad-spectrum activity [113], which is unfortunately rapidly degraded by proteases [114]. Liposomal encapsulation protected and preserved the antimicrobial activities of IK8. To form hydrogels, a solution of four-arm PEG maleimide was mixed with IK8-encapsulated liposomes and lipid-coated gold nanorods (AuNRs). This solution was mixed thoroughly with PEG dithiol to form the hydrogels. The PEG hydrogel was chosen because gelation occurs through a Michael-type reaction, which allows for tunable gelation rates and mechanical properties, and because of its high permeability by small hydrophilic molecules, it should not interfere with AMP release kinetics [115,116,117]. The fabricated hydrogels were irradiated with near infrared light (860 nm) at 1.8–2.8 W/cm^2^ for 10 min, which enabled the control of the saturation temperature between 50 and 65 °C. Upon heating to 55 °C, about 65% of encapsulated IK8 and AuNRs are released from a 5% hydrogel, causing significant bacterial killing against *P. aeruginosa* and *S. aureus*, with no cytotoxicity. In this study, hydrogels containing AuNRs alone, IK8 alone, and their combination were analyzed, and it was found the presence of AuNRs exhibited better photothermal activity and enhanced the bactericidal activity of IK8 against bacterial biofilms. Based on these findings, this hydrogel has excellent potential for the development of advanced wound dressings that can provide light-triggered and highly effective antimicrobial therapy. However, in vivo studies with animal models and the further optimization of the hydrogel formulation for long-term stability and biocompatibility are required before any practical clinical application can be developed.

### 4.2. Release of AMPs in Response to pH

**Figure 3:**
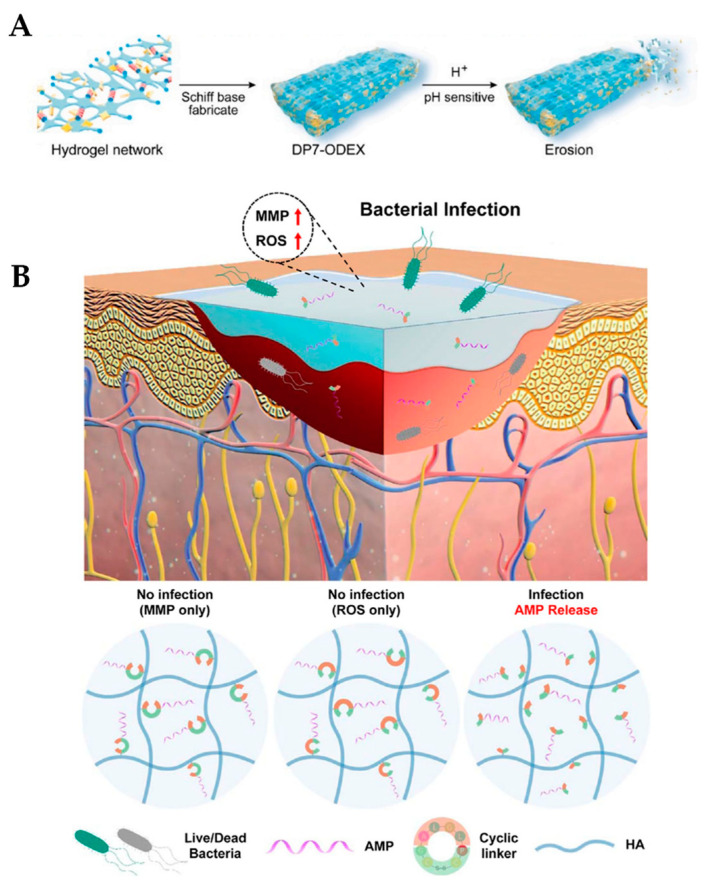
Release mechanisms for AMP-loaded hydrogels. (**A**) Structure of DP7-ODEX hydrogel. The hydrogel network is fabricated by Schiff base chemistry, forming DP7-ODEX. When exposed to low pH, the hydrogel erodes, releasing antimicrobial contents. Reprinted with permission from Ref. [118]. Copyright 2022, Elsevier. (**B**) Schematic representation of the Ad-HA hydrogel conjugated to KR12 (AMP) as an injectable wound coating. The cyclic linker is responsive to both reactive oxygen species (ROS) and matrix metalloproteinases (MMPs) and will only release AMP in the presence of both, during bacterial infection. Reprinted with permission from Ref. [128]. Copyright 2023, American Chemical Society.

The second AMP release mechanism occurs when the pH of the environment changes, causing the release of antimicrobial cargo. Wu and coworkers hypothesized that a pH-sensitive hydrogel could release antimicrobial agents in response to the weakly acidic wound environment in a controlled manner, leading to faster wound-healing and reduced bacterial load [118]. The regions surrounding bacterial infections are typically mildly acidic due to the secretion of lactic acid by the invading bacteria. A pH-sensitive hydrogel was formed by combining oxidized dextran (ODEX) and a peptide called DP7 (VQWRIRVAVIRK). DP7 is a cationic AMP with broad antimicrobial activity that displays synergistic effects with various antibiotics [119,120]. ODEX cross-linked hydrogels are made by mixing a 6% DP7 solution with a 10% dextran-aldehyde in a 1:1 ratio and allowing time for gelation (DP7-ODEX hydrogels). The DP7-ODEX hydrogel forms via pH-sensitive Schiff base reversible covalent bonds, in which DP7 serves as a linker and forms a porous network with ODEX. The Schiff base linkage is broken down in the presence of protons in the environment, as in an acidic environment (Figure 3A). For synergy studies, the 3rd-generation cephalosporin ceftazidime was premixed with DP7 and then combined with the ODEX solution to form CAZ-DP7-ODEX hydrogels. The antimicrobial properties of the hydrogels were analyzed both in vitro and in vivo. Three different pathogenic bacteria were used, *P. aeruginosa*, *S. aureus*, and *E. coli.* The DP7-ODEX hydrogels had superior killing efficacy against all three species compared to control hydrogels. Moreover, when combined with ceftazidime, CAZ-DP7-ODEX hydrogels displayed an additive or synergistic effect against a collection of 27 multidrug-resistant *P. aeruginosa* isolates. Furthermore, the use of these hydrogels in both normal and diabetic mouse models showed faster wound-healing rates and the more complete regeneration of dermis and epidermis skin layers. In addition, the hydrogels were well-tolerated and did not cause any adverse effects in the mice. These results strongly suggest that pH-sensitive hydrogels made from dextran and pH-sensitive peptides have the potential to be an effective treatment for multidrug-resistant bacterial-infected wounds. While promising so far, this study only used a small sample size of mice, which limits the generalizability of the findings. The long-term safety and efficacy of the pH-sensitive hydrogels are worth being further investigated.

Wei et al. used a pH-sensitive hydrogel to prepare a system for the treatment of chronically infected wounds which regulates both the inflammatory response, through the AMPs and enhances collagen deposition and angiogenesis to promote wound-healing, through the addition of platelet-rich plasma (PRP) [121]. Like Zhang et al., the pH-sensitive hydrogel was formed based on Schiff base chemistry using ODEX. The hydrogels were prepared by mixing equal volumes of 100 mg/mL ODEX with 25 mg/mL AMP-modified HA (HA-AMP). The AMP used in this study was cecropin (SWLSKTAKKLFKKIPKKIPKKRFPRPRPWPRPNMI-NH_2_), which has a broad antimicrobial spectrum of activity [122,123]. The mechanism of action of cecropin is the disruption of membrane integrity by changing the conformation of the cell membrane, resulting in the leakage of cell contents [124]. To prepare a PRP-loaded hydrogel, PRP was added to a 3.125% solution of HA-AMP prior to mixing with an equal volume of ODEX, to create ODEX/HA-AMP/PRP hydrogels. Both the ODEX/HA-AMP and ODEX/HA-AMP/PRP hydrogels displayed good antimicrobial activity against *E. coli*, *S. aureus*, and *P. aeruginosa* without any cytotoxicity. Spectroscopy was used to monitor AMP release over a span of 14 days. The ODEX/HA-AMP hydrogel released 20% of the AMP on the first day and afterwards had a continuous, sustained released, with 10% AMP remaining on day 14. Next, the release kinetics of the growth factors PDGF, TGF-β, and EGF from the hydrogel were measured using ELISA. The release of each growth factor showed a linear cumulative release for the first 7 days, after which the release rate slowed down. This suggests that the hydrogel provides a stable supply of growth factors during the wound-healing period. Finally, using a diabetic mouse model, ODEX/HA-ADH/PRP hydrogels promoted the skin-healing of infected wounds but were not completely effective in inhibiting inflammation. All things considered, the ODEX/HA-AMP/PRP hydrogels were effective at accelerating wound-healing, lowering the bacterial burden, and balancing inflammatory cell infiltration. Therefore, these hydrogels are promising for the treatment of severely infected wounds, including problematic diabetic foot ulcers.

### 4.3. Enzymatic Release of AMPs

The third mechanism of AMP release is the enzymatic cleavage of AMPs that are tethered to a hydrogel surface. These enzymes may be host or bacterial in nature. Obuobi and coworkers synthesized AMP-loaded DNA hydrogels based on the high binding affinity between cationic AMPs and polyanionic DNA nanostructures [125]. The AMP L12 is a synthetic peptide (LKKL) with strong broad-spectrum activity against both susceptible and multidrug-resistant bacteria [126]. Hydrogels were formed by mixing a linker and Y-scaffold nanostructure solutions, heating and gradually cooling to 25 °C over two hours. Various concentrations of L12, from 125 to 250 μM, were then gently mixed with the nanostructures to ensure uniform drug dispersion and prevent precipitation. The resulting L12-loaded DNA hydrogels were biocompatible and had significant activity against methicillin-resistant and susceptible *S. aureus* and *E*. *coli*. As the release of AMPs are triggered by the enzymatic secretion from the bacteria, the DNA hydrogels were tested in the presence and absence of DNase I. The L12 had a half-life of 3 h when subjected to 10 U/mL of DNase I, with a complete release by 12 h, but the half-life could be reduced to 30 min if exposed to more DNase I. These results show that the release of loaded AMPs can be controlled in response to DNA degradation. Ex vivo antimicrobial studies using porcine skin infected with *S. aureus* showed a 4-log reduction in bacterial numbers within 4 h for groups treated with L12-loaded DNA hydrogels. These results demonstrate the potential for treating cutaneous *S. aureus* infections. As an application of these DNA hydrogels is wound-healing, their ability to modulate inflammation was examined by monitoring TNF-α, a pro-inflammatory cytokine released after macrophage activation. In the presence of lipopolysaccharide (LPS), a component of the Gram-negative bacterium cell wall and powerful immunogen, macrophages significantly increased TNF-α release. However, LPS-stimulated cells co-treated with the DNA hydrogels resulted in significant reduction in TNF- α production, thereby demonstrating anti-inflammatory properties and potential to accelerate wound-healing. To confirm the in vitro findings, DNA hydrogels were applied on mice excision wounds and healing properties evaluated for ten days. Wound healing was evaluated by measuring changes in the residual wound area and re-epithelialization rates. There were faster wound-healing rates in the DNA-treated hydrogels than control hydrogels. In addition, the formation of granulation tissues was significantly greater in hydrogel-treated mice than the controls, indicating that these hydrogels could be used for deep or chronic wounds. These DNA hydrogels have high potential, but there may need to be additional optimizations required to increase the stability of the hydrogels, as after 24 h without enzyme, more than 50% of the AMP was already released.

Patients with diabetic wounds are frequently exposed to bacterial infections, which causes the damaged skin tissue to heal more slowly due to hyperglycemia [127]. Jeong and colleagues developed an injectable, controlled AMP-releasing hydrogel for the treatment of infected diabetic wounds with high stability and less toxicity [128]. The hydrogels were prepared by modifying HA with cyclodextrin (CD) to synthesize HA-CD and adamantane (Ad) to form Ad-HA through amidation and esterification, respectively. Equal amounts of HA-CD and Ad-HA were mixed for 15 s to form hydrogels. The KR12 AMP (KRIVQRIKDFLR-NH_2_), the shortest alpha helical derivative of the human cathelicidin LL-37 AMP with antimicrobial activity [129,130], was conjugated to Ad-HA hydrogels through a cyclic peptide linker comprising a reactive oxygen species (ROS) and matrix metalloprotease (MMP) cleavable sequence to form Ad-HA-AMP hydrogels. The infection-responsive cyclic linker helps in the controlled release of the AMP only in the presence of both MMP and ROS during infection, which minimizes AMP toxicity (Figure 3B). The Ad-HA-AMP hydrogels displayed enhanced antibacterial activity against *E. coli* and *S. aureus* and increased serum stability compared to KR12 alone. Strikingly, the soluble KR12 lost its antibacterial activity in 72 h, while the hydrogel-embedded KR12 maintained its activity. KR12 is short and is easily degraded by proteolytic enzymes in the body. In an in vitro cytocompatibility assay on fibroblast cells, Ad-HA-AMP hydrogel did not release AMP in the absence of ROS and MMP, thereby inducing cell proliferation and minimizing cytotoxicity. Moreover, in an in vivo diabetic wound-healing mouse model, the AMP-containing hydrogels showed enhanced and accelerated wound-healing activity in infected mice. In contrast, the controls delayed wound-healing and thus did not cure the bacterial infections. The controlled release of the AMP from these hydrogels minimizes side effects, treats infection, and displays efficient wound-healing. These supramolecular hydrogels have a high potential to be developed into new wound coatings that would be particularly useful for the treatment of chronic wounds, especially in diabetic patients.

### 4.4. Nanoparticles as Drug Delivery Systems

The use of nanoparticles as drug delivery systems has been increasingly explored in recent years [131]. Among the many nanomaterials available, mesoporous silica nanoparticles (MSNs) are particularly attractive because they are biocompatible and capable of anchoring to multiple different functional groups. In addition, MSNs have a large surface area and a large porous volume, which improve the ability to load therapeutic cargo [132,133]. In a recent study by Ma and coworkers, the objective was to design an MSNs modified with an ovotransferrin-derived AMP that could act as a mechanism to interact with the bacteria and produce antimicrobial activities against them [134]. The amphipathic AMP OVTp12 was derived from egg white ovotransferrin, which displayed excellent antimicrobial activity through the disruption of the bacterial cell membrane [135]. MSNs were synthesized by heating a mixture of hexadecyltrimethyl ammonium bromide and NaOH. Tetraethyl orthosilicate was added to precipitate the MSNs. OVTp12 was coupled to the MSNs using NHS and EDC chemical crosslinking agents, which were mixed in solution for 24 h to form MSNs@OVTp12 particles. In this study, gentamicin was also included and mixed with the MSNs@OVTp12 particles at room temperature for 24 h, forming MSNs@OVTp12@Gen particles. In a weakly acidic environment, the cargo was continuously released over 72 h. The presence of OVTp12 on the surface allowed for effective interaction with *E. coli* and enhanced bacterial killing. MSNs@OVTp12@Gen was found to have effective properties for both attracting and adhering to bacteria and bactericidal activity. In vivo, MSNs@OVTp12@Gen enhanced overall survival and more effectively treated *E. coli* infections in mice than MSNs@Gen or gentamicin treatment alone. In addition, these particles were found to decrease inflammatory cytokine release and were noncytotoxic in vivo. The MSNs@OVTp12@Gen designed and used in this study had the ability to tolerate weakly acidic environments and had a strong, bactericidal effect, which makes them promising for targeted antibiotic delivery to bacterial infection sites. The potential to incorporate MSNs into hydrogel matrices is exciting and should be further explored, as MSNs are bioactive, can improve mechanical properties of the hydrogel, and can release various types of cargo in a controllable manner [136]. Together, an MSN-hydrogel could be designed with superior antimicrobial and healing properties, while limiting the emergence of further resistance.

## 5. Conclusions, Challenges, and Perspectives

AMP-loaded hydrogels as antibacterial weapons are an attractive alternative solution to traditional antibiotics, which, in the face of the current antimicrobial resistance crisis, is of utmost importance. Exciting new antimicrobial hydrogel coatings are rapidly being investigated and developed. In this review, we highlighted recent work on AMP-loaded hydrogels from the last five years that has advanced the development of photopolymerizable, self-forming, and AMP-releasing hydrogels (Table 1). These studies clearly demonstrate that a wide variety of cationic or amphipathic AMPs retain good antibacterial activity when tethered on or within a hydrogel matrix. Overall, each of the newly studied hydrogels discussed here resulted in decreased cytotoxicity, improved antimicrobial killing, and, in many cases, enhanced wound-healing. It is important to note that the AMP concentrations of many of the studies presented were seemingly arbitrary. High AMP concentrations can be cytotoxic, so future AMP hydrogels could be designed with minimalist AMP concentrations that maximize bactericidal properties while minimizing unwanted cytotoxicity. One promising way to reduce the amount of total AMP is to explore the use of multiple AMPs that have synergistic effects. These interactions can be determined using a simple microbial growth technique called a checkerboard assay, where each AMP is serially diluted horizontally or vertically to examine combinatorial effects [137]. Reducing the amount of AMP required not only further reduces cytotoxic potential but also makes large-scale production more practical. In addition, using a combination of AMPs with different mechanisms of action may provide superior microbicidal activity and further reduce the possibility of the development of downstream AMP resistance.

The potential of these novel AMP-loaded hydrogel formulations to become clinically approved coatings is exciting and likely in the foreseeable future. However, there are multiple considerations and challenges that remain and must be considered before use in clinical practice becomes a reality. What features are the most important when designing a practical antimicrobial coating? The field agrees that potent, broad spectrum antimicrobial activity is important. In addition, little to no hemolytic or cytotoxicity is essential. However, there seems to be some disagreement on whether AMPs would be better suited in a controlled and prolonged release platform or whether they should be covalently tethered. AMP hydrogel self-assembly is guided by hydrophobic forces and π–π stacking or covalent crosslinking using polymers, such as chitosan [138,139,140]. One notable issue with self-assembling AMP hydrogels is the requirement for high concentrations of AMP, which may introduce off-target cytotoxicity and would increase manufacturing costs. In contrast, AMP hydrogels with a non-AMP backbone can include lower concentrations of AMP while still forming a hydrogel, thereby potentially circumventing limitations seen with self-assembling AMP hydrogels. As hydrogels can be functionalized with multiple bioactive molecules and combined with other nanoparticles, another challenge will be to determine the optimal ratio and compatibility of each component so that they produce synergistic effects.

Without a doubt, it is worthwhile to investigate and optimize different multifunctional hydrogel configurations to develop practical applications for clinical use. It will be important to continue to optimize and broaden the antimicrobial spectrum of activity. For example, a wound coating that features antimicrobial activity against many pathogens, including Gram-negative and -positive bacteria and skin infection-causing fungi, would be ideal. It is already possible to design hydrogels with excellent antimicrobial properties in combination with hemostatic, immunomodulatory, antithrombotic, and healing properties. The maximum number of beneficial factors that can be incorporated into a single hydrogel formulation are not currently known but the possibilities and combinations are limitless. The future may simply involve the production of highly specialized hydrogels tailored to specific medical needs to provide superior therapy. These antimicrobial hydrogels are broadly applicable to many fields, including wound dressings, catheters, biofilm-based medical device infections, dental coatings, and even contact lenses. One thing is certain—the future of AMP-loaded hydrogel technology for the treatment of infections is bright.

## Figures and Tables

**Table 1:** Summary of recently developed AMP hydrogels.

AMP(s)	Antimicrobial Activity	Studied	Unique Features/ Applications	Reference
Dhar5	*S. epidermidis*	In vitro	Surface coatings	Alves et al. [51]
	*P. aeruginosa*			
Battacin	*S. aureus*	In vitro	Topical antibacterial	De Zoysa et al. [54]
	*P. aeruginosa*		agents	
HHC-36	MRSA	In vitro		
	*S. epidermidis*	In vivo	Sprayable wound	Cheng et al. [56]
	*P. aeruginosa*		dressing	
	*E. coli*			
Tet 213	MRSA	In vitro	Sprayable wound	Annabi et al. [62]
	*E. coli*	In vivo	dressing	
WR and Bac2a	*S. aureus*	In vitro	Wound dressing	Liu et al. [65]
	*E. coli*	In vivo	coating	
RRWRVIVKW	*S. aureus*	In vitro	Wound dressing	Feng et al. [75]
	*E. coli*	In vivo	coating	
FKF	*P. aeruginosa*	In vitro		Azoulay et al. [82]
	*E. coli*	In vivo	Injectable wound	
	*A. baumannii*		dressing	
	*S. epidermidis*			
KK-11 and KKd-11	*S. aureus*	In vitro	Enzymatically stable	Guo et al. [88]
	*E. coli*		antimicrobial hydrogels	
PAF26	*S. aureus*	In vitro	Injectable antimicrobial	Cao et al. [89]
	*E. coli*		hydrogels	
	*C. albicans*			
NAVSIQKKK	*S. aureus*	In vitro	Biocompatible	Adak et al. [92]
	*E. coli*		antimicrobial hydrogels	
KK(SLKL)_3_KK	*S. aureus*	In vitro	Injectable wound	Suo et al. [94]
	*E. coli*	In vivo	dressing	
Tet 213	*S. aureus*	In vitro	Bone-forming	Yang et al. [95]
		In vivo	antibacterial hydrogels	
Jelleine-1	MRSA/MSSA	In vitro	Postoperative, adhesive	Zhou et al. [99]
	*E. coli*	In vivo	antibacterial hydrogels	
	*C. albicans*			
Piscidin-1	*A. baumannii*	In vitro	AMP-releasing with	Rezaei et al. [108]
			temperature	
IK8	*S. aureus*	In vitro	AMP-releasing with	Moorcroft et al. [112]
	*P. aeruginosa*		light	
DP7	*S. aureus*	In vitro		Wu et al. [118]
	*E. coli*	In vivo	AMP-releasing with pH	
	*P. aeruginosa*			
Cecropin	*S. aureus*	In vitro	AMP and PRP hydrogels	Wei et al. [121]
	*E. coli*	In vivo	for wound healing	
	*P. aeruginosa*			
L12	MRSA/MSSA	In vitro	Anti-inflammatory	Obuobi et al. [125]
	*E. coli*	In vivo	antimicrobial hydrogels	
KR12	*S. aureus*	In vitro	Antimicrobial hydrogels	Jeong et al. [128]
	*E. coli*	In vivo	for diabetic wounds	
OVTp12	*E. coli*	In vitro	Antibacterial NPs	Ma et al. [135]
		In vivo	for wound infections	

MRSA: methicillin-resistant *S. aureus*, MSSA: methicillin-susceptible *S. aureus*, NP: nanoparticle.

## Data Availability

Data sharing not applicable.
